# Combined Effects of Dual-Scale Modified Surface with Micro- and Nanostructures on the Cellular Biocompatibility, Osteoinduction, and Antibacterial Properties of Titanium Implants

**DOI:** 10.3390/jfb16050157

**Published:** 2025-04-28

**Authors:** Shaheer Maher, Nenad L. Ignjatović, Miloš Lazarević, Sanja Petrović, Andrijana Žekić, Dusan Losic

**Affiliations:** 1 Faculty of Pharmacy, Assiut University, Assiut 71526, Egypt; shaheer.maher@aun.edu.eg; 2 Institute of Technical Sciences of the Serbian Academy of Sciences and Arts, Knez Mihailova 35/4, PAK 104105, 11000 Belgrade, Serbia; 3 School of Dental Medicine, University of Belgrade, Dr Subotica 8, 11000 Belgrade, Serbia; milos.lazarevic@stomf.bg.ac.rs (M.L.); sanja.petrovic@stomf.bg.ac.rs (S.P.); 4 Faculty of Physics, University of Belgrade, Studentski trg 12, 11000 Belgrade, Serbia; andrijana@ff.bg.ac.rs; 5 School of Chemical Engineering, The University of Adelaide, Adelaide, SA 5005, Australia

**Keywords:** titanium, titanium implants, osseointegration, nanostructures, microstructures, additive manufacturing, selective laser melting, hydrothermal process

## Abstract

Titanium implants are widely used in biomedical applications due to their excellent mechanical properties and biocompatibility. However, implant-associated bacterial infections and suboptimal osseointegration remain significant challenges. Recent studies have demonstrated that the interplay between micro- and nanostructures can enhance both biocompatibility and antibacterial properties. This study explores the synergistic effects of hierarchical and dual surface topography on Ti surfaces with micro- and nanostructures to demonstrate their ability to promote cellular biocompatibility and osteoinduction while simultaneously inhibiting bacterial colonization. The combination of selective laser melting (SLM) to create micro-structured surfaces and hydrothermal processes is used to generate distinctive nanopillar structures. By integrating nanoscale features that mimic the extracellular matrix with microscale topographies that influence cellular responses, we achieve a balance between enhanced osseointegration and antimicrobial performance. The physicochemical properties of these dual-scale topographies are characterized through cellular assays using dental pulp stem cells (DPSCs), demonstrating sustained support for long-term cell viability (above 78% in MTT and NR assays (*p* < 0.05), low levels of LDH release, and high levels of cellular migration) and osteoinduction (statistically significant (*p* < 0.0001) ALP activity increase and higher levels of calcified matrix deposition, upregulation of *ALP* and *OCN* genes compared with smooth surface topographies). Their antibacterial properties against *S. aureus* and *E. coli* showed a significant reduction (*p* < 0.05) in bacterial attachment and biofilm formation. Our findings highlight the potential of multi-scale surface modifications as a promising strategy for next-generation titanium implants, paving the way for improved clinical outcomes in orthopedic and dental applications.

## 1. Introduction

Numerous studies have been performed aimed at eliminating the inherent limitations and improving the performance of current titanium (Ti) implants with enhanced antibacterial properties and osseointegration capabilities for dental and orthopedic applications [1,2,3,4,5]. These studies indicate that modifying the physicochemical properties of implant surfaces, such as surface charge, hydrophobicity, roughness, and structures, has shown promise in improving both cell attachment and bacterial resistance.

One notable approach involves the electrochemical anodization of titanium to form titania nanotubes (TNTs) to make these unique nanostructures on the Ti surface [6,7]. These nanotubes not only exhibit antibacterial properties but also facilitate the localized delivery of antimicrobial agents [3,8,9]. Another strategy introduced by Ivanova et al. involves mimicking the surface features of insect wings (e.g., cicadas or dragonflies) with nanoscale sharp structures capable of mechanically disrupting bacterial cells by inducing membrane rupture, effectively eliminating bacterial attachment and biofilm formation without the need for antibiotics [10,11,12]. This approach offers a cost-effective and scalable method for generating antibacterial surfaces on titanium implants. From these studies, hierarchical topographies on the implant surface, which combine microscale roughness with nanoscale features, have been introduced in many studies showing great promise in improving biocompatibility and antibacterial properties. Microscale structures contribute to mechanical interlocking with bone tissue, promoting osseointegration, while nanoscale features mimic the extracellular matrix, facilitating cellular interactions and reducing bacterial adhesion [1,3,5,13,14,15,16].

These dual-scale modifications offer a novel approach to achieving a balance between enhanced osteogenic potential and antibacterial efficacy. However, the impact of various surface topographies—such as micro- and nano-features—on bone tissue formation remains a topic of ongoing debate, largely due to variations in fabrication methods and structural inconsistencies. While micro-topographical surfaces have been shown to improve bone fixation and in vivo pullout strength, recent studies suggest that nanoscale features, which mimic the hierarchical structure of bone, may further enhance osteoblast activity, increase cell attachment, and promote bone formation [17,18,19,20]. Nano-textured surfaces have demonstrated excellent biocompatibility by facilitating protein adsorption, promoting apatite formation, and providing an optimal environment for cell proliferation and anchoring [17,19,20,21]. The processes of osseointegration and bacterial infection are intricately linked. The “race to the surface” hypothesis suggests that bacteria often colonize the implant surface before human cells can attach, due to factors such as the surgical environment, implant handling, and the presence of contaminants [21,22]. Therefore, developing an implant surface that balances the promotion of bone cell attachment with resistance to bacterial colonization and biofilm formation is essential for improving implant longevity and functionality.

Over the past decade, additive manufacturing (AM) technologies, such as 3D printing, have gained considerable attention for their potential to revolutionize the production of titanium-based medical implants [23]. AM offers unique advantages, including design flexibility and the ability to fabricate implants with complex geometries that closely resemble bone structures [24]. Moreover, AM allows for patient-specific implants that can be produced rapidly and tailored to individual anatomical needs, reducing the need for mass production and excess inventory. Among various AM techniques, selective laser melting (SLM), also known as direct metal printing (DMP), stands out as a highly effective method for manufacturing titanium implants. SLM enables the fabrication of titanium and titanium-alloy implants with precisely controlled properties, reduces material waste, and lowers overall production costs [2,24,25,26]. In our previous work, we explored several approaches to enhance the performance of 3D-printed titanium for biomedical applications, focusing on surface modifications via electrochemical treatments, chemical etching, and hydrothermal processes [13,14,15,27,28,29]. Our findings demonstrated that nanopillar structures significantly improved antibacterial properties by mechanically disrupting bacterial cell membranes while supporting osteoblastic cell growth [13,27]. AM of Ti implants presents a promising solution for developing the next generation of low-cost, customizable, and efficient Ti alloy implants for dental and orthopedic applications, and their further advancements in surface engineering are essential to fully realize their potential, enhancing their performance and biocompatibility.

**Figure 1:**
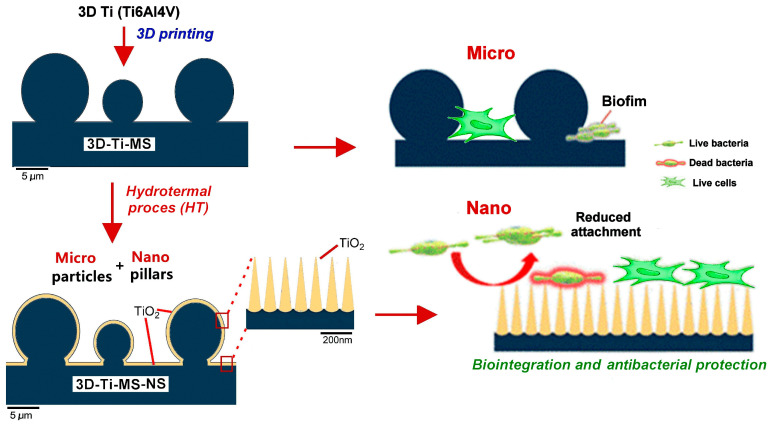
Schematic diagram of the cellular biocompatibility and antibacterial protection of titanium alloys with dual micro- and nano-topography.

Building upon this promising development, the present study aims to further investigate the biocompatibility and antibacterial properties of titanium alloys with dual micro- and nano-topography fabricated via AM (SLM). Specifically, we examine how different surface features at varying scales, such as micro-smooth, micro-rough, and nanopillar structures, can affect both cellular behavior (osteoblast attachment and proliferation) and bacterial attachment, biofilm formation, and bactericidal activity. This comprehensive study on the interaction of stem cells and bacteria on fabricated nano- and microstructures on Ti surfaces fabricated by a combined SLM and hydrothermal method is illustrated in Figure 1. Dental pulp stem cells (DPSCs) are employed as model cells to assess the integration potential of these surfaces, which are particularly relevant to the emerging field of 3D-printed dental implants [30,31]. Additionally, we use Escherichia coli and Staphylococcus aureus to evaluate the antibacterial efficacy of the implants. The findings of this study provide valuable insights into the interplay between implant surface topography, cell behavior, and bacterial colonization, contributing to the development of advanced medical implants with optimized biocompatibility and antibacterial properties using AM technologies.

## 2. Materials and Methods

### 2.1. Fabrication of 3D-Ti Implants

Titanium implants (termed 3D-Ti-MS) were fabricated using titanium alloy (Ti6Al4V) powder (Titanium grade 5, TLS Technik GmbH & Co. Spezialpulver, Bitterfeld, Germany) in the forms of square discs (1.5 × 1.5 cm^2^) via selective laser melting (SLM) using a ProX 200 Production 3D Printer (Phenix Systems PXM, Alpharetta, GA, USA). The SLM system was equipped with a 300 W laser (wavelength 1070 nm, operating at 50% power) and processed under an inert argon atmosphere. Detailed information on the fabrication process and the average particle diameter of the powder can be found in our previous work [28]. Following fabrication, any residual powder particles were removed from the wafer surfaces by sonication in acetone for 10 min. The resulting implants, featuring a micro-smooth surface, will be referred to as “Ti” throughout the paper.

### 2.2. Surface Modification

To make a nanostructured surface on a 3D-Ti-MS, hydrothermal surface modification was performed, adapting previously described HT procedures [32,33,34]. Briefly, 3D-Ti-MS discs were submerged in 50 mL of 1 M NaOH (Chem-Supply, Adelaide, Australia) in a Teflon-lined vessel, which was then placed inside a stainless-steel autoclave. The samples were hydrothermally (HT) treated for 4 h at 160 °C. After the HT process, the autoclave was removed from the oven and allowed to cool to room temperature. The discs were rinsed three times with Milli-Q water to remove any remaining NaOH and were then air-dried at room temperature overnight. The samples were subsequently annealed in a tube furnace at 300 °C for 3 h under atmospheric conditions. These HT-treated samples are referred to as 3D-Ti-MS-NS substrates.

Prior to cell and bacterial studies, the prepared 3D-Ti-MS and 3D-Ti-MS-NS samples were immersed in Milli-Q water for 2 weeks to ensure complete removal of any residual chemicals from the fabrication process.

### 2.3. Materials Characterizations

High-resolution imaging of various surfaces was performed using a Focused Ion Beam (FIB)-Scanning Electron Microscope (SEM, FEI Helios Nanolab 600 Dual Beam, Thermo Fisher Scientific, Scoresby, Australia), operated at 10 kV. Surface chemical characterization was carried out via Energy Dispersive X-ray Spectroscopy (EDX, Oxford Ultim Max Large Area SDD EDS detector, Oxford Instruments, Concord, MA, USA). Prior to SEM imaging, all wafers were coated with a 5 nm layer of platinum. The crystallinity of the surfaces was analyzed by X-ray diffraction (XRD, Rigaku MiniFlex 600, Osaka, Japan), operating at 40 kV and 15 mA, using Cu Kα radiation. XRD spectra were recorded at an angular scan rate of 10° per minute over the 30°–80° range under ambient conditions.

Water contact angle (WCA) measurements were conducted using the sessile drop method at room temperature, employing a Tension Theta optical tensiometer (KSV Instruments, Helsinki, Finland) equipped with an automated stage, droplet dispenser, and digital camera. A 2 µL drop of Milli-Q water (resistivity 18.2 MΩ·cm) was used, and measurements were taken at three distinct locations on each sample, which was prepared in triplicate—three separate wafers. The images were analyzed with OneAttension software (version 3.2, Biolin Scientific, Gothenburg, Sweden). To minimize the influence of environmental factors on WCA measurements, prepared samples were stored in a clean, airtight desiccator immediately after fabrication. This approach prevents contamination and moisture adsorption, which can significantly alter surface properties and affect CA values. Furthermore, CA measurements were conducted promptly after removing the samples from the desiccator, thereby reducing exposure to ambient conditions such as humidity and airborne contaminants that could impact the results.

### 2.4. In Vitro Studies

#### 2.4.1. Cell Cultures

Dental pulp stem cells (DPSCs) were isolated using an explant culture method from dental pulp tissues of immature permanent third molars obtained from healthy donors (*n* = 3, aged between 18 and 21 years) who provided written informed consent. The study was approved by the Ethical Committee (No. 36/26, 19 November 2021) at the School of Dental Medicine, University of Belgrade. The cells were cultured in complete growth medium consisting of DMEM/F12, supplemented with 10% fetal bovine serum (FBS) and 1% antibiotic/antimycotic (all from Gibco, ThermoFisher, Waltham, MA, USA). Cultures were maintained at 37 °C in a humidified 5% CO_2_ atmosphere and passaged routinely when they reached 80% confluence. The medium was refreshed every 2–3 days. The cells were characterized as DPSCs based on criteria outlined in a previous study [35]. Only cells between the 3rd and 5th passage were used for the experiments. Before cell seeding, alloy wafers were sterilized by immersion in 70% (*v*/*v*) ethanol for 5 min, followed by air-drying in a Class II biohazard hood for 3 h and three rinses in sterile water. Pure titanium (Ti) wafers were used as controls in all experiments. Triplicate substrates were placed in a single well of a 6-well tissue culture plate.

#### 2.4.2. MTT and Neutral Red Assays

Two distinct methods, MTT and Neutral Red (NR) assays, were used to evaluate cytotoxicity: indirect cytotoxicity assay and direct cytotoxicity assay.

For indirect cytotoxicity assays, Ti-based wafers (Ti, 3D-Ti-MS, and 3D-Ti-MS-NS) were first immersed in complete growth medium for 7 days. Subsequently, DPSCs were seeded at a density of 5 × 10^3^ cells/mL into 96-well plates (Corning, New York, NY, USA) and exposed to the supernatants from the respective wafers (Ti, 3D-Ti-MS, or 3D-Ti-MS-NS) for 1, 3, or 7 days. After the designated incubation periods, 100 µL of MTT solution (0.5 mg/mL) was added to each well and incubated for 3 h at 37 °C. For the NR assay, 150 µL of 1× NR Staining Solution (Sigma-Aldrich, St. Louis, MO, USA) was added to each well and incubated for 4 h at 37 °C. Following incubation, the medium was aspirated, and 100 µL of dimethyl sulfoxide (DMSO, Sigma-Aldrich) for MTT or 150 µL of NR eluent (96% ethanol: dH_2_O: CH_3_COOH, 50:49:1) for NR assay was added per well. The plates were then shaken at 250 RPM for 15 min. Optical density (OD) was measured at 570 nm using an automated microplate reader (RT-2100c, Microplate Reader, Rayto, Shenzhen, China). Cells without treatment served as the positive control. The percentage of viable cells was calculated using the following formula:

Viable cells = (OD (sample) − OD (blank))/(OD (control) − OD (blank)) × 100.

For the direct cytotoxicity assays, Ti wafers were placed in individual wells of a 12-well tissue culture plate (Corning, New York, NY, USA), and DPSCs were seeded directly onto the wafer surfaces at a density of 5 × 10^3^ cells/mL. The cells were incubated for 1, 3, or 7 days, with cell viability assessed using both MTT and NR assays, as described above. For the MTT assay, 500 µL of MTT solution was added, while for the NR assay, 500 µL of 1× NR Staining Solution was used. After the incubation period, the culture medium was removed, and either DMSO or NR eluent (depending on the assay) was added. The OD of the plates and the percentage of viable cells were calculated as described above.

#### 2.4.3. Lactate Dehydrogenase (LDH) Assay

Cytotoxicity was also assessed by measuring the release of extracellular lactate dehydrogenase (LDH) using the CyQUANT™ LDH Cytotoxicity Assay (Catalog no. C20300, ThermoFisher, Waltham, MA, USA). DPSCs were seeded at a density of 2 × 10^4^ cells/well in 12-well plates with Ti, 3D-Ti-MS, or 3D-Ti-MS-NS substrates and incubated for 1, 3, or 7 days. At each time point, the LDH release was quantified according to the manufacturer’s protocol. Absorbance was measured at 490 nm using a microplate reader (RT-2100c Microplate Reader, Rayto, Shenzhen, China).

#### 2.4.4. Wound Healing Assay

DPSCs were seeded at a density of 2 × 10^4^ cells/well in 12-well plates, with titanium samples placed in each well. Once the cells reached confluence, a scratch was made using a 200 µL pipette tip along the edge of the Ti sample (Figure S1). The wells were then washed with phosphate-buffered saline (PBS, Gibco, ThermoFisher, Waltham, MA, USA) to remove cell debris. The closure of the scratched area was monitored over 48 h at 5 min intervals using a CytoSMART™ Lux2 camera (CytoSmart Technologies, Amsterdam, The Netherlands) [32]. CytoSMART software (version 1.0) was used to quantify the healing area (µm^2^). Representative images were extracted at 0, 12, 24, 36, and 48 h to track the migration process.

#### 2.4.5. Alkaline Phosphatase (ALP) Activity Assay

To evaluate functional alkaline phosphatase (ALP) activity, 4 × 10^5^ DPSCs were seeded in 12-well plates (1 mL per well) with different titanium wafers and cultured in human StemMACSOsteoDiff Media (osteogenic medium, OM, Miltenyi Biotec, Tokyo, Japan) for 7 days. After the incubation period, ALP activity was assessed using a commercially available pNPP Alkaline Phosphatase assay kit (Sigma-Aldrich, St. Louis, MO, USA). The OD was measured at 405 nm using a microplate reader (RT-2100C Microplate Reader, Rayto, Shenzhen, China).

#### 2.4.6. Osteogenic Differentiation and Alizarin Red Staining

One day after seeding DPSCs (5 × 10^4^ cells/well) on Ti, 3D-Ti-MS, or 3D-Ti-MS-NS substrates in 12-well plates, OM was added. The cells were incubated for 7, 14, and 21 days in the osteogenic media and wafers, with OM changes every 3 days. The effect of 3D-Ti-MS and 3D-Ti-MS-NS on mineralized nodule formation was evaluated by Alizarin Red S (ARS) staining. At each time point (7, 14, and 21 days), the cultures were fixed with 4% neutral formalin for 15 min and stained with 2% ARS (Sigma-Aldrich). To quantify mineralization, ARS-bound dye was extracted by incubating the cultures with 250 µL of 1% hydrochloric acid in 70% ethanol for 20 min. The absorbance of the extracted dye was measured at 450 nm using a microplate reader (RT-2100c, Microplate Reader, Rayto, Shenzhen, China) [36]. Cells cultured in OM only served as the control.

#### 2.4.7. Real-Time Quantitative Polymerase Chain Reaction (qPCR)

DPSCs were seeded on wafers and cultured for 21 days to allow osteogenic differentiation. Total RNA was then extracted from the cells using Trizol reagent (Invitrogen, Thermo Fisher Scientific, Waltham, MA, USA). Complementary DNA (cDNA) was synthesized using the RevertAid First Strand cDNA Synthesis Kit (Thermo Fisher Scientific), following the manufacturer’s instructions. RT-qPCR analysis was performed on the cDNA using the same RevertAid kit to assess the expression of osteogenic markers *ALP* and *OCN*. Specific primers for these markers are listed in Table S1. GAPDH was used as the reference housekeeping gene. Fold induction values were calculated using the 2^−ΔΔCt^ method [34].

#### 2.4.8. Scanning Electron Microscope (SEM) Imaging of Cells

DPSCs (5 × 10^4^ cells per well) were seeded onto different wafer substrates and incubated for 4 days in complete growth medium in 12-well plates. The morphology of the cells attached to the substrates was assessed using scanning electron microscopy (SEM) [37]. After the 4-day incubation, the samples were gently rinsed with Milli-Q water and fixed with 2.5% (*v*/*v*) glutaraldehyde (Sigma). Following fixation, the samples were rinsed twice with Milli-Q water. The cells were then dehydrated through a graded series of ethanol solutions (30%, 50%, 70%, 90%, and 100% *v*/*v*) for 10 min each, with an additional 10 min treatment in 100% ethanol. After dehydration, the samples were immersed in hexamethyl disilazane (HMDS) (Sigma) for 10 min. The samples were left to air-dry and then sputter-coated with gold using a JFC 1100 ion sputter for imaging. High-resolution images were captured using a JEOL JSM-840A SEM instrument at an acceleration voltage of 30 kV.

### 2.5. Antibacterial Activity Studies

#### 2.5.1. Bacterial Growth

*Escherichia coli* ATCC 25922 and *Staphylococcus aureus* ATCC 25923 (Microbiologics KWIK-STIK, Manassas, VA, USA) were revived from frozen stocks stored at −80 °C by incubation at 37 °C for 24 h, as outlined in Table S2. After activation of the reference strains, 3–4 colonies of *S. aureus* and *E. coli* were transferred to separate Brain Heart Infusion (BHI) broth (HIMEDIA, Maharashtra, India) and incubated for an additional 24 h at 37 °C in aerobic conditions. The bacterial suspensions were centrifuged (3000 RPM for 5 min). The supernatant was discarded, and the pellet of each suspension was resuspended in sterile PBS to achieve a turbidity corresponding to the 0.5 McFarland standard (≈10^8^ bacterial cells/mL). Each suspension was further diluted in enriched BHI broth to obtain two suspensions (one of *S. aureus* and the other of *E. coli*) with a final concentration of approximately 10^6^ bacterial cells/mL.

#### 2.5.2. Biofilm Formation

Monomicrobial bacterial biofilms were formed by adding 1 mL of each described bacterial suspension (≈10^6^ bacteria cells/mL) to the surface of prepared Ti (control), 3D-Ti-MS, and 3D-Ti-MS-NS samples placed in 24-well microplates. The plates were incubated statically under aerobic conditions at 37 °C. For each microorganism, monomicrobial biofilm was formed on three groups of materials: Ti (*n* = 3), 3D-Ti-MS (*n* = 3), and 3D-Ti-MS-NS (*n* = 3).

##### SEM Visualization of Monomicrobial Biofilms

For visualization of the formed monomicrobial biofilm of *S. aureus* and *E. coli* on 3D-Ti-MS and 3D-Ti-MS-NS samples, scanning electron microscopy was performed. After 24 h of biofilm formation, wafers were removed from the medium, gently washed with sterile PBS to remove unattached bacterial cells, and fixed in 2.5% glutaraldehyde (Sigma, Aldrich) for 48 h. Subsequently, wafers were dehydrated by applying a series of solutions of 3% acetic acid, 3% acetic acid and 25% ethanol, 3% acetic acid and 50% ethanol, and 70% ethanol, according to a previously described fixation method [38].

##### Determination of Colony Forming Units (CFU) from Medium Around the Samples

After biofilm formation on the samples, twelve 10-fold serial dilutions of the medium surrounding the samples were seeded on solid media: Columbia agar with 5% sheep blood for *S. aureus* and Endo agar for *E. coli*. The plates were incubated at 37 °C under aerobic conditions, and CFUs were counted after 24 h.

##### MTT Assay of Medium Around the Samples

After the removal of the wafers for MTT analysis, 50 µL of the medium from each well was transferred to eight wells of a new 96-well microplate. The same volume (50 µL) of MTT solution was added to each well containing the medium. The plates were incubated in lightproof conditions at 37 °C for 3.5 h under static aerobic conditions. After incubation, 100 µL of dimethyl sulfoxide (DMSO) was added to each well, and the plates were shaken at 250 rpm for 15 min at 37 °C in the dark. The absorbance of the resulting colored product, which reflects bacterial viability, was measured at 540 nm using a spectrophotometer.

### 2.6. Statistical Analysis

Statistical analysis and generation of graphical images were conducted using GraphPad Prism version 9 (GraphPad Software, Inc., San Diego, CA, USA). All results in this study are presented as the mean ± standard deviation (SD) of at least three independent experiments. Statistical comparisons were made using one-way analysis of variance (ANOVA) followed by Tukey’s multiple comparisons test, with a single pooled variance. The normality of data distribution was assessed using the Kolmogorov–Smirnov test. Statistical significance was set at *p* < 0.05 for all comparisons. All in vitro experiments were performed in triplicate and repeated at least twice.

## 3. Results

### 3.1. Surface Morphology and Physico-Chemical Properties of Fabricated Combined Micro- and Nanostructures

**Figure 2:**
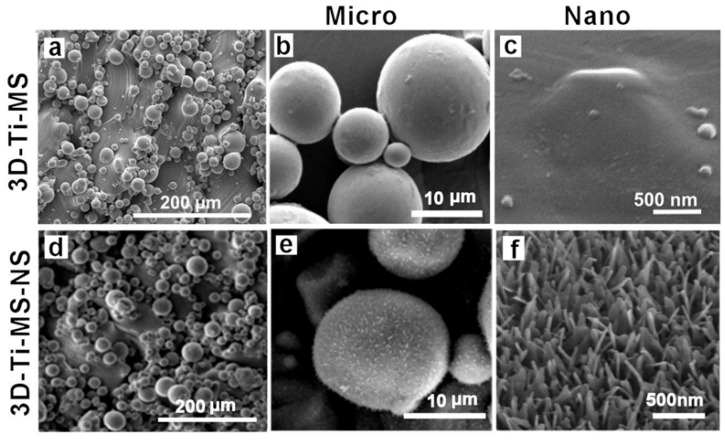
SEM images showing the top surface of (**a**–**c**) a 3D-Ti-MS substrate with spherical microparticle topography fabricated by the SLM process showing typical structures of microparticles and a flat surface between these particles; (**d**–**f**) a 3D-Ti-MS-NS substrate that retains microstructure topography, the surface of which is covered by nanopillar structures generated by the hydrothermal process with 1 M NaOH.

The topographic morphology and structural characteristics of fabricated titanium (Ti) wafers with micro- and nanostructures obtained by SEM imaging are summarized in Figure 2. The surface of the 3D-Ti-MS fabricated using SLM equipment exhibits randomly distributed microspheres, which result from the partial melting of Ti alloy powder during the SLM process (Figure 2a,b). During the SLM process, layers of Ti alloy powders with spherical microparticles are melted by the laser beam to generate microparticles inside the bulk structure on the top surface. These microspheres, which have a diameter ranging from 5 μm to 20 μm, are randomly distributed on the surface, with different inter-distances and very strong adhesion on the surface with an average surface roughness (Ra) of 17 ± 3 µm.

Apart from the microparticles, the peak and valley structures of the surface appeared smooth, exhibiting no nanostructures. After fabrication, prepared 3D-Ti-MS samples were sonicated in acetone to remove any loosely adhered particles an ensure the stability of the topographic features during subsequent processing. Apart from the microspheres, the surface exhibits a smooth topography with peaks and valleys but lacks nanostructures (Figure 2c). Following the fabrication of 3D-Ti-MS, hydrothermal treatment was applied to generate 3D-Ti-MS-NS nanostructures, which have typical topography presented in Figure 2d–f. The images show that these nanostructures are composed of arrays of nanopillars with wide bases and sharp ends with a length in the range of 400–500 nm and inter-distances between 150–250 nm.

**Figure 3:**
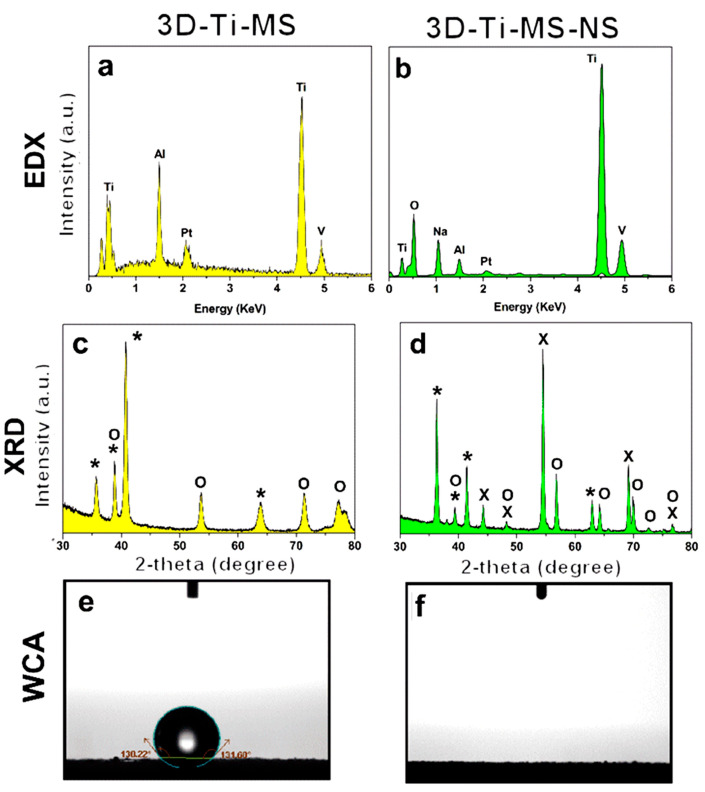
Chemical and physical characterization and surface properties of prepared 3D-Ti-MS and 3D-Ti-MS-NS substrates demonstrating (**a**,**b**) EDX spectra showing surface chemical composition, (**c**,**d**) XRD patterns with peaks corresponding to Ti, anatase TiO_2_, and Na_2_Ti_3_O_7_ (*—Ti, O—TiO_2_, X—titanate), and (**e**,**f**) wetting properties by water contact angle (WCA).

The physical and chemical properties of 3D-Ti-MS and 3D-Ti-MS-NS were characterized using EDX, XRD, and WCA measurements, the results of which are presented in Figure 3. EDX spectra of all samples displayed the typical peaks of Ti, Al, and V that are expected, as they were fabricated from titanium alloy (Ti_6_Al_4_V) powders by the SLM printing process (Figure 3a,b). In addition, an Na peak is detected for 3D-Ti-MS-NS, which could correspond to sodium titanate (Na_2_Ti_3_O_7_) that is created during the HT process. The crystal structure of the prepared 3D-Ti-MS and 3D-Ti-MS-NS samples assessed by XRD showed the distinct diffraction peaks presented in Figure 3c,d. The high crystalline structure of all samples was confirmed through sharp, high-intensity diffraction peaks. All samples showed Ti peaks (JCPDS 44-1294), while anatase TiO_2_ peaks (JCPDS 21-1272) and (Na_2_Ti_3_O_7_) (JCPDS 31-1329) were evident after hydrothermal processing.

The measurement of the water contact angle (WCA) on the prepared substrates presented in Figure 3e,f revealed distinct differences in surface wettability based on the surface modifications. The 3D-Ti-MS exhibited hydrophobic characteristics, with WCAs of 133 ± 1 degrees, respectively, consistent with the typical hydrophobic behavior of metal surfaces. On the other hand, the 3D-Ti-MS-NS surface displayed superhydrophilic behavior, where the water droplet spread instantly, preventing accurate WCA measurement. This dramatic increase in wettability can be primarily attributed to the formation of sodium titanate (Na_2_Ti_3_O_7_) during the hydrothermal treatment. Sodium titanate is known to enhance surface hydrophilicity, likely by increasing the surface energy and creating a more porous surface structure.

### 3.2. DPSCs Viability and Integration Studies on Combined Micro- and Nanostructures

#### 3.2.1. MTT Assay

**Figure 4:**
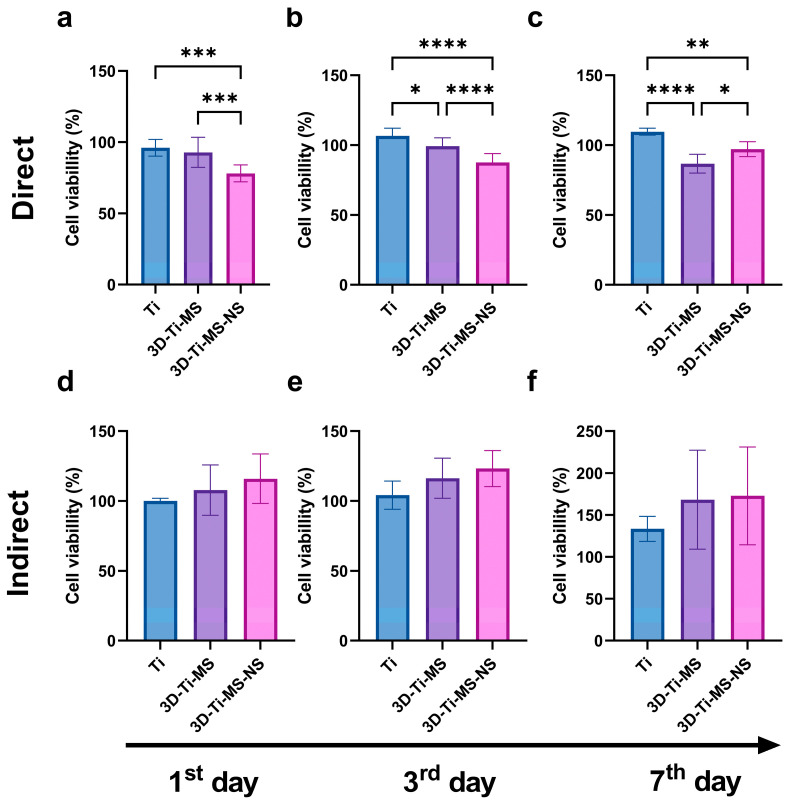
MTT assay of dental pulp stem cells (DPSCs) on prepared samples including Ti, 3D-Ti-MS, and 3D-Ti-MS-NS showing (**a**) direct MTT assay 1-day treatment; (**b**) direct MTT assay 3-day treatment; (**c**) direct MTT assay 7-day treatment; (**d**) indirect MTT assay 1-day treatment; (**e**) indirect MTT assay 3-day treatment; (**f**) indirect MTT assay 7-day treatment; * *p* ≤ 0.05; ** *p* ≤ 0.01; *** *p* ≤ 0.001; ****p ≤ 0.0001.

The results of the MTT assay, showing DPSCs viability on prepared samples including Ti, 3D-Ti-MS, and 3D-Ti-MS-NS with microspheres and combined microspheres and nanopillar topography over 7 days, are summarized in Figure 4. Results indicate that cells in direct contact with the different Ti materials did not exhibit a significant loss in viability (1st day: Ti group 96.1% (±5.9); 3D-Ti-MS group 92.9% (±10.5); 3D-Ti-MS-NS group 78.1% (±5.9); 3rd day: Ti group 106.6% (±5.5); 3D-Ti-MS group 99.3% (±5.9); 3D-Ti-MS-NS group 87.6% (±6.3); 7th day: Ti group 109.6% (±2.5); 3D-Ti-MS group 86.9% (±6.8); 3D-Ti-MS-NS group 97.2% (±5.3)), with cell viability remaining above 70%, indicating good support for cell growth. On day 1, the cell viability of both Ti and 3D-Ti-MS surfaces was similar (Figure 4a). However, by day 3, 3D-Ti-MS showed a reduction in cell viability compared with Ti (Figure 4b), and by day 7, the cell viability of 3D-Ti-MS was lower compared with 3D-Ti-MS-NS (Figure 4c).

This trend confirms that surface topography influences cell viability, as previously reported in many other studies [3,5,7,15,17,18,19,28,29,39,40]. The highest cell viability was observed in the Ti group (smooth surface), where it exceeded 100% after 3 and 7 days of culture. Interestingly, the 3D-Ti materials (3D-Ti-MS and 3D-Ti-MS-NS) demonstrated a significantly better effect on cell viability (1st day: Ti group 100.2% (±1.9); 3D-Ti-MS group 107.8% (±18.1), 3D-Ti-MS-NS group 116.0% (±17.8); 3rd day: Ti group 104.2% (±10.1); 3D-Ti-MS group 116.3% (±14.3), 3D-Ti-MS-NS group 123.2% (±12.9); 7th day: Ti group 113.4% (±14.9); 3D-Ti-MS group 168.2% (±59.1), 3D-Ti-MS-NS group 172.8% (±58.3)) after indirect treatment (indirect MTT, Figure 4d–f), although this difference was not statistically significant when compared with the Ti group.

#### 3.2.2. Neutral Red Assay

**Figure 5:**
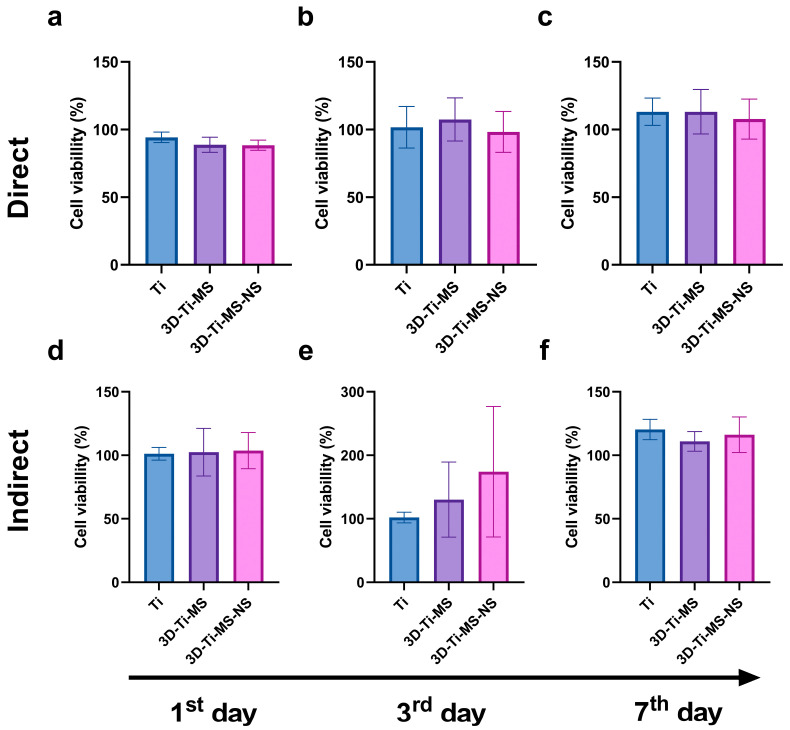
NR assay with dental pulp stem cells (DPSCs) on prepared samples including Ti (control), 3D-Ti-MS, and 3D-Ti-MS-NS showing (**a**) direct NR assay 1-day treatment; (**b**) direct NR assay 3-day treatment; (**c**) direct NR assay 7-day treatment; (**d**) indirect NR assay 1-day treatment; (**e**) indirect NR assay 3-day treatment; (**f**) indirect NR assay 7-day treatment.

The results of the Neutral Red (NR) assay of DPSCs on prepared substrates, including Ti, 3D-Ti-MS, and 3D-Ti-MS-NS, are presented in Figure 5. Cells in direct contact with all these surfaces showed no significant loss in viability (1st day: Ti group 94.4% (±3.8); 3D-Ti-MS group 88.8% (±5.6); 3D-Ti-MS-NS group 88.4% (±4.4); 3rd day: Ti group 101.7% (±15.4); 3D-Ti-MS group 107.4% (±15.9); 3D-Ti-MS-NS group 98.2% (±15.1); 7th day: Ti group 113.1% (±10.1); 3D-Ti-MS group 113.2% (±16.5); 3D-Ti-MS-NS group 107.7% (±14.8)) and did not indicate cytotoxicity for any of the materials tested. Additionally, no significant differences in viability were observed between the different substrates. Similar results were obtained using the indirect method to assess cell viability (1st day: Ti group 101.2% (±5.1); 3D-Ti-MS group 102.4% (±18.8); 3D-Ti-MS-NS group 103.6% (±14.3); 3rd day: Ti group 102.0% (±8.5); 3D-Ti-MS group 130.2% (±49.1); 3D-Ti-MS-NS group 174.2% (±92.8); 7th day: Ti group 120.3% (±8.1); 3D-Ti-MS group 111.1% (±7.8); 3D-Ti-MS-NS group 116.2% (±13.9)), where no significant differences in viability were noted between the substrates after 7 days of incubation with DPSCs. Therefore, the NR assay aligns with MTT results and confirms that all tested surfaces are biocompatible and can support DPSCs growth after implantation into the body.

#### 3.2.3. LDH Assay

**Figure 6:**
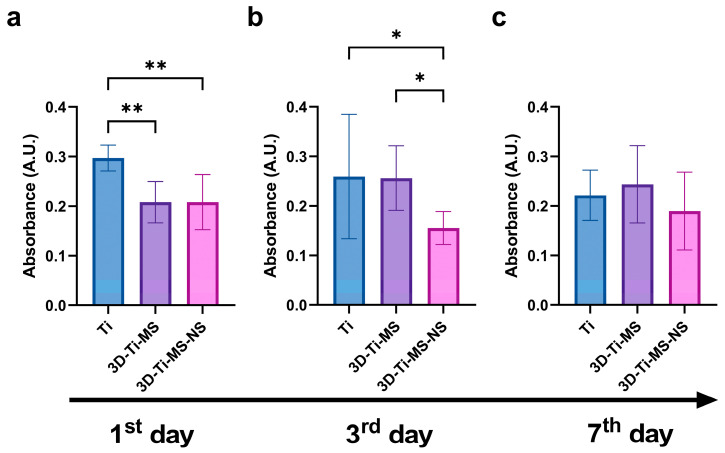
LDH activity of dental pulp stem cells (DPSCs) cultured on Ti (control), 3D-Ti-MS, and 3D-Ti-MS-NS substrates measured at (**a**) the 1st day; (**b**) the 3rd day; and (**c**) the 7th day of direct contact with materials; * *p* ≤ 0.05; ** *p* ≤ 0.01.

LDH activity in the DPSC culture media was measured as an indicator of cell membrane integrity during 7 days of interactions with Ti (control), 3D-Ti-MS, and 3D-Ti-MS-NS substrates and is presented in Figure 6. The 3D-surfaced materials showed significantly lower levels of LDH after one and three days of culturing with DPSCs compared with the Ti control (Figure 6a,b). These results suggest that Ti substrates caused considerable cell membrane damage, whereas 3D-Ti-MS-NS supported the maintenance of DPSCs membrane integrity. Based on the cell viability and LDH assay results, 3D-Ti-MS-NS emerges as a promising candidate for implant surfaces, particularly in the critical initial days following implant insertion, a period that plays a pivotal role in determining the success of the implant [41].

**Figure 7:**
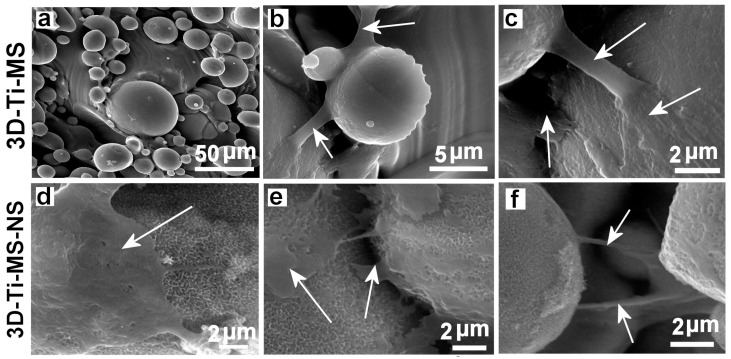
SEM images showing the dental pulp stem cells (DPSCs) interacting with (**a**–**c**) 3D-Ti-MS with spherical microparticle topography and (**d**–**f**) 3D-Ti-MS-NS substrate that combined microstructure topography covered but nanopillar structures generated by hydrothermal process. Arrows show the filopodia projection of the cells in the direction of bridging structures on the surface.

The interaction of DPSCs with 3D-Ti-MS and 3D-Ti-MS-NS substrates was evaluated using SEM imaging, with the results presented in Figure 7. These images reveal differences in cellular interactions between the two substrate types. On the 3D-Ti-MS surfaces, most cell propagation occurs at the interface between the microspheres and the surface, with cells attempting to bridge these gaps (Figure 7a–c, arrows). On the 3D-Ti-MS-NS substrates, which feature both micro- and nanopillar topographies, such interconnections are also present; however, the majority of cells are observed lying directly on the microsphere surfaces or in the spaces between them (Figure 7d–f). These findings suggest a stronger interaction of DPSCs with the 3D-Ti-MS-NS substrate, indicating improved biointegration properties as a result of synergetic interaction between micro- and nanostructures that are proposed by other studies [13,14,15,29].

#### 3.2.4. Wound Scratch Healing Assay

**Figure 8:**
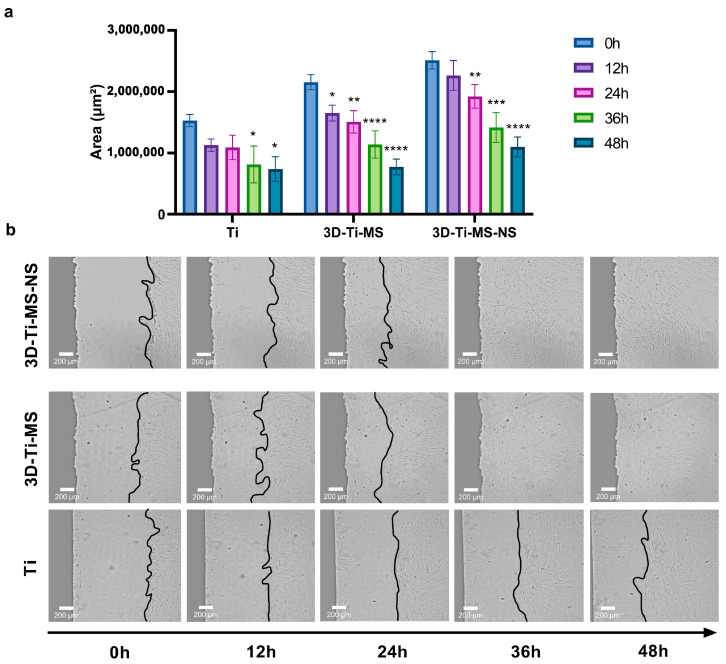
Wound scratch healing assay on Ti (control), 3D-Ti-MS, and 3D-Ti-MS-NS substrates on cell migration showing (**a**) the graph of closing of the scratch area during 48 h; (**b**) representative micrographs at 0, 12, 24, 36, and 48 h after the scratch; * *p* ≤ 0.05; ** *p* ≤ 0.01; *** *p* ≤ 0.001; **** *p* ≤ 0.0001 (vs. 0 h group). (the real-time of the cells movements are presented in Videos S1–S3).

To assess the impact of the prepared samples, including Ti (control), 3D-Ti-MS, and 3D-Ti-MS-NS substrates, on cell migration, the cell monolayers were “wounded” near the material borders and allowed to heal over 48 h, the results of which are summarized in Figure 8. Results show that interaction with the 3D-printed materials resulted in a significantly faster cell migration rate when compared with Ti (0 h) (Figure 8a,b and Supplementary Videos S1–S3), indicating that 3D-Ti-MS and 3D-Ti-MS-NS surfaces promoted rapid cell healing. These results suggest that both 3D-Ti-MS and 3D-Ti-MS-NS could better support cell growth and provide the long-term attachment necessary for successful permanent implants compared with smooth Ti surfaces. This enhanced performance can be attributed to the ability of the micro- and combined micro- and nano-textured surfaces to promote cell adhesion and proliferation, as previously confirmed in our and other studies [13,14,15,29].Results showed that both bone cells and fibroblasts exhibited strong anchoring through the formation of extended pseudopodia and focal adhesions, which can contribute to more effective osseointegration.

#### 3.2.5. Alkaline Phosphatase (ALP) Activity Assay

**Figure 9:**
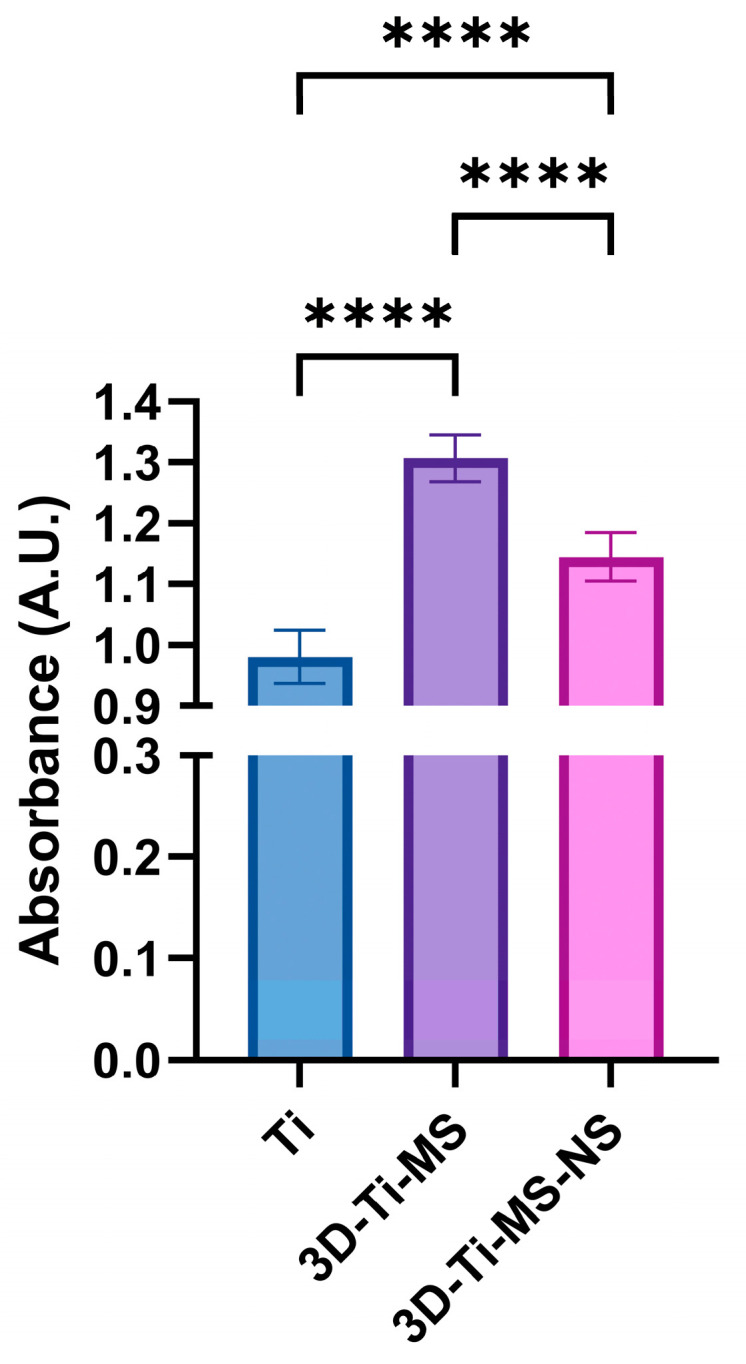
ALP activity of DPSCs cultured in osteogenic media measured on Ti (control), 3D-Ti-MS, and 3D-Ti-MS-NS substrates, **** *p* ≤ 0.0001.

The interaction of DPSCs cells cultured in the osteogenic medium over 7 days with Ti (control), 3D-Ti-MS, and 3D-Ti-MS-NS substrates is presented in Figure 9. Notably, the interaction of DPSCs with the 3D materials with micro and combined micro and nanostructures resulted in significantly higher ALP activity (*p* ≤ 0.0001) compared with Ti control samples. The highest ALP activity was observed in the cells in contact with 3D-Ti-MS, with microstructures that indicate that these structures effectively promote mineralization in DPSCs, a process that is closely associated with cellular differentiation. This process is more intensive compared with 3D-Ti-MS-NS with combined micro- and nanostructures.

#### 3.2.6. Alizarin Red S Quantification

**Figure 10:**
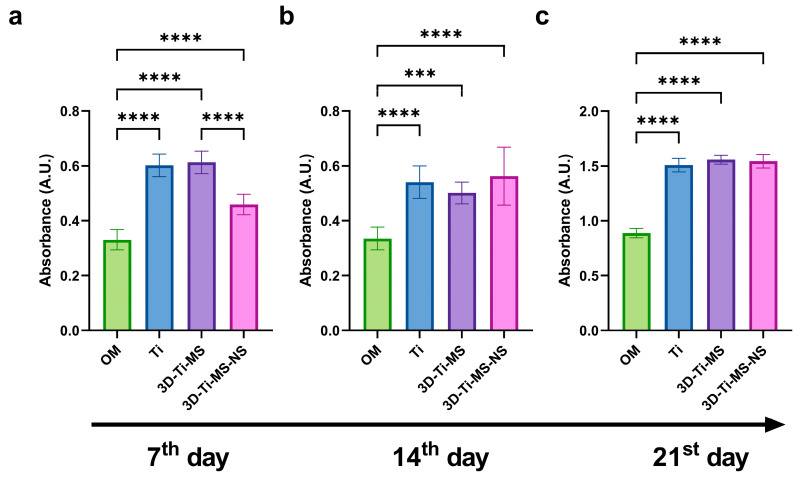
Alizarin Red S staining quantification of DPSCs upon osteogenic differentiation on Ti, 3D-Ti-MS, and 3D-Ti-MS-NS substrates after (**a**) 7, (**b**) 14, and (**c**) 21 days of osteoinduction; OM—cells cultured only in osteogenic medium. *** *p* ≤ 0.001; **** *p* ≤ 0.0001.

Alizarin Red S staining of cells upon 7, 14, and 21 days of osteogenic differentiation on different Ti surfaces, including Ti, 3D-Ti-MS, 3D-Ti-MS-NS, and control (cells cultured only with osteogenic medium—OM), are presented in Figure 10. Cells cultured on flat and non-modified Ti surfaces and 3D-Ti-MS-NS exhibited a higher level of calcified matrix deposition compared with the control group after just 7 days of osteoinduction. These findings are consistent with our previous studies and align with the ALP activity results [29].

Alizarin Red staining revealed enhanced mineralization in cells exposed to the materials compared with those cultured with osteogenic media alone over periods of 7, 14, and 21 days, with a significant difference (*p* ≤ 0.001) compared with the control. This increased mineralization is indicative of cellular differentiation. During this process, an extracellular matrix (ECM) rich in collagen is initially formed, followed by the deposition of hydroxyapatite (HAP)-like mineralized protein matrix, which is in agreement with previous studies [42,43].

#### 3.2.7. qPCR

**Figure 11:**
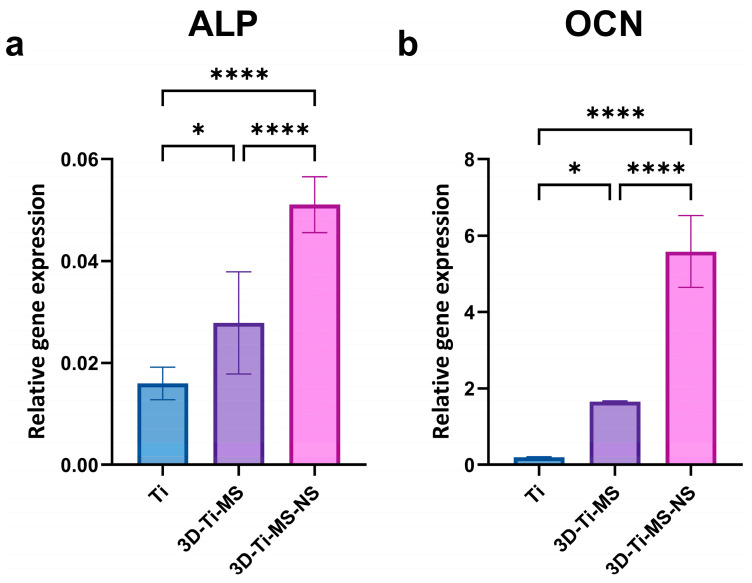
qPCR analysis of gene expression after 21 days of osteoinduction of cells in contact with Ti (control), 3D-Ti-MS, and 3D-Ti-MS-NS substrates showing (**a**) *ALP* and (**b**) *OCN* osteogenic markers; * *p* ≤ 0.05; **** *p* ≤ 0.0001.

qPCR analysis and gene expression after 21 days of osteoinduction of cells in contact with Ti (control), 3D-Ti-MS, and 3D-Ti-MS-NS substrates are presented in Figure 11. Gene expression plays a crucial role in DPSC differentiation into osteoblasts, and this study showed that cells cultured on titanium materials with micro- and combined micro- and nanostructures exhibited significantly (*p* ≤ 0.05) higher expression of key osteogenic genes, such as alkaline phosphatase (*ALP*) and osteocalcin (*OCN*), after 21 days of osteoinduction.

### 3.3. Bacterial Interaction Studies on Combined Micro- and Nanostructures

**Figure 12:**
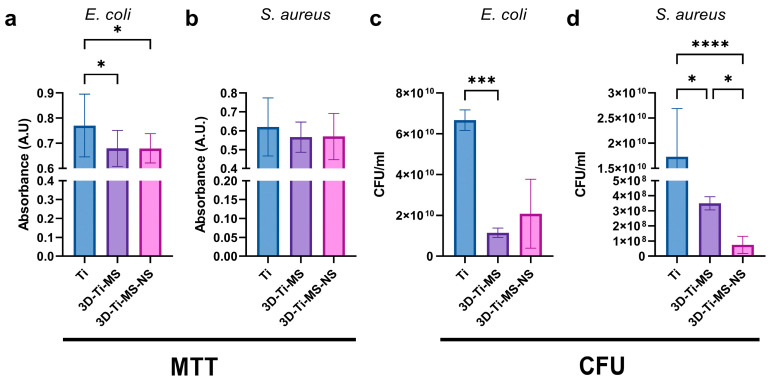
Bacterial (**a**,**b**) MTT and (**c**,**d**) CFU assays in media around Ti (control), 3D-Ti-MS, and 3D-Ti-MS-NS substrates; * *p* ≤ 0.05; *** *p* ≤ 0.001; **** *p* ≤ 0.0001.

The number of bacterial cells in the medium around Ti (control), 3D-Ti-MS, and 3D-Ti-MS-NS substrates assessed using CFU and MTT assays after 24 h of incubation with *E. coli* and *S. aureus* are presented in Figure 12. CFUs of both bacteria in the medium around the samples were lower for 3D-Ti-MS than around control samples. The number of *S. aureus* around samples was lower for both types of 3D-Ti samples compared with the control, presenting that nanopillars showed better results against Gram-positive bacteria (Figure 12c,d). In addition, the MTT assay results, as a measure of bacterial metabolism, were consistent with the CFU findings. Bacterial viability was significantly lower (*p* ≤ 0.0001) for *E. coli* around the 3D-Ti-MS and 3D-Ti-MS-NS plates compared with the smooth Ti (Figure 12a). The same trend was noted in the *S. aureus* group, however, without reaching a statistically significant difference (Figure 12b).

**Figure 13:**
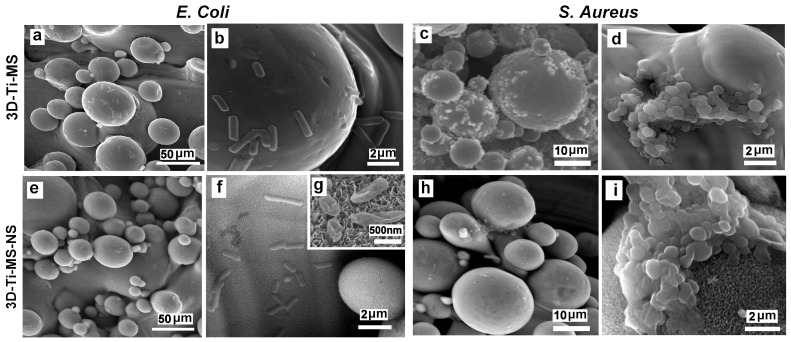
SEM images showing the interaction of 3D-Ti-MS substrate with spherical microparticle topography with (**a**,**b**) *E. coli* bacteria and (**c**,**d**) *S. aureus* and 3D-Ti-MS-NS substrate that combines microstructure topography covered with nanopillar structures with (**e**–**g**) *E. coli* and (**h**,**i**) *S. aureus*.

The interaction of 3D-Ti-MS and 3D-Ti-MS-NS substrates with micro- and combined micro- and nano-topography and two bacteria, *E. coli* and *S. aureus*, is visualized by SEM imaging showing a series of SEM images summarized in Figure 13. These results show that more *E. coli* and *S. aureus* bacteria cells are observed attached to 3D-Ti-MS compared with 3D-Ti-MS-NS. In both cases, the formation of biofilms is observed on the crevices of microspheres where large numbers of bacteria have settled down and agglomerated. In the case of 3D-Ti-MS-NS, a lower number of bacteria was seen on the microsphere surface and the surface between microspheres, indicating the ability of nanopillar structures to reduce *E. coli* and *S. aureus* viability compared with 3D-Ti-MS substrates. Moreover, SEM images confirmed the nanopillar structures induced membrane damage inflicted on attached *E. coli* bacterial cells, as seen in Figure 13g, showing the destroyed bacteria body. Overall, it can be concluded that 3D-Ti-MS-NS could significantly reduce the occurrence of bacterial attachment and eradicate those bacterial cells that do attach to the surface compared with smooth Ti substrates.

## 4. Discussion

### 4.1. Projected Design of Micro- and Nano-Topography

To investigate the biocompatibility and antibacterial properties of titanium alloys with dual micro- and nano-topography fabricated via AM (SLM) in this study, we successfully fabricated these model implant substrates that have different surface features at varying scales, such as micro-smooth and micro-rough with spherical particle morphology, which are additionally modified with nanostructured surfaces with distinctive sharp nanopillar structures. Our morphological analysis confirmed that these 3D-printed Ti alloy substrates have characteristic microparticle topographies. Previous studies showed many advantages of these microstructures, which improve the biointegration of these emerging 3D-printed implants in orthopedic medicine and dentistry [44]. The sharp nanostructures in the form of nanopillars are successfully generated on our 3D-Ti-MS substrates (Figure 2). The HT process, involving oxidation and etching of the 3D-Ti-MS wafers followed by the growth of sodium titanate (Na_2_Ti_3_O_7_) crystals on the surface, gradually made the distinct nanopillar structures. One of our hypotheses was that these nanopillar structures demonstrate strong antibacterial properties, primarily attributed to their ability to mechanically disrupt the membranes of bacterial cells and eventually kill bacteria [45,46].The second hypothesis posited that, despite the antibacterial properties of these surfaces, they would maintain cellular biocompatibility.

The ability to produce wafers with varying hydrophobic and hydrophilic properties is of great importance, as the interaction between implants, cells, and bacteria is significantly affected by surface hydrophilicity. Previous studies have indicated that hydrophilic surfaces enhance the repulsion between bacterial cells and the implant surface while simultaneously reducing the hydrophobic interactions between the bacterial cell membrane and the implant, leading to a decrease in bacterial attachment on the surface [47].The nanostructures formed on the 3D-Ti-MS-NS surface, particularly the nanopillars, contributed to the superhydrophilic nature of the surface (Figure 3f). The wettability of the surface is a critical feature influencing the interaction between cells or bacteria and the implant surface. Previous studies showed that hydrophilicity causes a significant reduction in bacterial cell attachment and film formation on the surface of implants due to inhibition of hydrophobic interactions, promoting the repulsion between the surface and bacteria [15,30]. This effect is particularly important for implants, as bacterial colonization can lead to infection or Ti implant failure. In addition, combining nanostructures with hydrophilicity can enhance protein adsorption and improve osseointegration [42,47].

### 4.2. Influence of Surface Topography on Cellular Biocompatibility

The MTT, NR, and LDH assay results provided insights into the biocompatibility of titanium-based materials with different surface topographies, namely Ti, 3D-Ti-MS, and 3D-Ti-MS-NS. The findings indicate that all tested substrates supported DPSCs viability, with no significant decrease in cell viability, suggesting that these materials are generally biocompatible and provide a favorable environment for cell growth. The viability of cells in direct contact with the various surfaces remained high, with no essential differences in DPSCs viability between the Ti, 3D-Ti-MS, and 3D-Ti-MS-NS groups (Figure 4a–c, Figure 5a–c and Figure 6). This is a promising outcome, indicating that all these surface modifications support cell growth effectively. Additionally, the indirect MTT and NR viability test, which simulates the conditions after implantation where the cells are not directly in contact with the surface but exposed to the materials microenvironment, showed no significant differences in DPSCs viability across the substrates over the 7-day incubation period. The 3D-Ti-MS-NS surface, which features both microspheres and nanopillars, showed a more favorable outcome in terms of cell membrane integrity compared with 3D-Ti-MS (LDH assay, Figure 6). The presence of nanopillars, which potentially mimic natural extracellular matrix features and likely contributed to enhanced cell attachment and proliferation, possibly by providing additional surface area for cell interaction. Previous studies have indicated that surface roughness and microstructure can affect cell adhesion and proliferation, which might explain the observed statistical differences in cell viability over time.

Cell morphology analysis by SEM (Figure 7) revealed that the surface roughness and topography of the 3D-Ti-MS and 3D-Ti-MS-NS materials facilitate better contact between cells and the implant surface, leading to stronger focal adhesions and more effective cell spreading. In our previous studies, both osteoblasts and fibroblasts showed improved anchorage to roughened titanium surfaces, forming multiple, longer pseudopodia and focal cell-matrix adhesions [28,29]. These interactions are key to the successful integration of the implant into the bone, contributing to more effective osseointegration over time.

Our study additionally assessed the material biocompatibility using a wound scratch healing assay (Figure 8). This method involved the mechanical disruption of cell monolayers at the material interface, resulting in a standardized “wound,” which was then monitored for migration and closure. In our study, this assay indicated whether the materials hinder or promote cell movement, which is essential for implant-tissue integration. The results indicated that while cell migration on 3D-Ti (3D-Ti-MS and 3D-Ti-MS-NS) materials was slightly higher than on the smooth Ti surface during the first 24 h, the difference was not statistically significant. However, between 24 and 48 h, cells in contact with the 3D surfaces showed a significantly greater migration rate. This enhanced cell migration is a critical factor in the initial stages of osseointegration, as it supports the healing process and encourages the attachment and proliferation of bone cells around the implant. The rapid migration observed on 3D-Ti surfaces suggests that these materials may promote better early-stage cell attachment and proliferation, which is important for the long-term success of dental implants. Overall, based on cell viability, cell morphology, and migration, 3D-Ti-MS-NS demonstrates the potential to be a suitable candidate for implantable surfaces, as it supports cellular attachment, growth, and migration, minimizing cellular damage.

### 4.3. Influence of Surface Topography on Parameters of Osseointegration

The ultimate goal of dental implants is to provide long-term functionality and promote rapid bone healing, which begins with osseointegration as the result of the interaction between osteoblasts and the implant surface. This is achieved through osseointegration, a critical process where bone cells interact with and attach to the implant surface. For this reason, we investigated the impact of different titanium surface topographies on the osteogenic differentiation of DPSCs, measured by ALP activity, ARS staining, and qPCR analysis of osteogenesis-related genes. Our results demonstrated that treatment with 3D-Ti materials significantly enhanced ALP activity in DPSCs (Figure 9), with the most pronounced increase observed in cells cultured on 3D-Ti-MS. This suggests that the 3D-Ti surface topography is highly effective in promoting osteoblastic differentiation and mineralization, processes essential for bone formation. The activity of osteoblasts and their ability to form new bone is influenced by the enzyme alkaline phosphatase. In mineralized tissues, ALP plays a crucial role in promoting the formation of hydroxyapatite crystals by increasing the local concentration of inorganic phosphate while simultaneously reducing the extracellular levels of pyrophosphate, an inhibitor of mineral formation [5]. ALP is a well-established biomarker of osteoblastic activity and plays a central role in bone formation by facilitating the mineralization process.

The enhanced ALP activity observed in the 3D-Ti-MS group supports our third hypothesis that micro/nanoscale surface modifications can improve the osteogenic potential of titanium materials. This increased ALP activity is indicative of improved cellular differentiation, which is critical for the success of dental implants and other bone-related applications. The results from this study are consistent with previous research showing that microstructured surfaces can enhance osteoblastic differentiation and mineralization [1,5,7,17,40,48].

The enhanced mineralization observed in cells cultured on the 3D titanium surfaces was further confirmed by Alizarin Red S quantification (Figure 10), which highlighted a greater deposition of mineralized matrix compared with cells treated only with osteogenic media. This difference was statistically significant across all time points (7, 14, and 21 days), suggesting that the surface topography of titanium plays an important role in accelerating osteo-differentiation and matrix mineralization. These findings are consistent with our previous studies, where we demonstrated that surface features such as roughness and micro/nanostructures can improve osteogenic differentiation and mineralization [1,5,7,17,40,42,48,49].

However, the ALP activity assay and ARS staining assessed osteogenic differentiation at a functional level. For that reason, we performed qPCR analysis (Figure 11) of *ALP* and *OCN* to provide additional mechanistic insights. Upregulated *ALP* and *OCN* gene expression confirmed that DPSCs were undergoing robust osteogenic differentiation at both the functional and transcriptional levels. Upregulated *ALP* and *OCN* gene expression suggests that differentiation is transcriptionally regulated, ensuring that high enzymatic activity and mineralization are not just transient effects but are driven by osteogenic gene activation. ALP is a marker of early osteoblast differentiation [50], while OCN is a late-stage marker primarily involved in the mineralization process [49]. The upregulation of both these genes indicates that the titanium materials, particularly the 3D surface structures, are not only biocompatible but also enhance osteoinduction of DPSCs. This is consistent with our earlier findings, where we demonstrated that micro/nanoscale surface modifications on titanium enhanced osteogenic differentiation and mineralization in vitro [29,42,46]. Further research, including in vivo studies, would be beneficial to confirm these findings and explore the long-term effects of these materials on osteointegration.

### 4.4. Influence of Surface Topography on Biofilm Formation and Antibacterial Properties

The ability of biomaterial surfaces to influence bacterial colonization and biofilm formation is a crucial factor in determining the success of implants, particularly in preventing infection and promoting long-term integration with the host tissue [51,52,53]. In this study, we evaluated the bacterial colonization on titanium surfaces with different topographies, such as 3D-Ti-MS and 3D-Ti-MS-NS substrates, by assessing bacterial cell counts through CFU and MTT assays and following SEM imaging. Interestingly, the bacterial load was higher on the smooth Ti surface than on 3D-Ti-MS and 3D-Ti-MS-NS substrates. This suggests that while the 3D surfaces may promote bacterial attachment in direct contact, they may also have a greater propensity for bacterial killing or inhibition of bacterial growth in the surrounding area. The indirect bacterial killing observed in the 3D-Ti-MS-NS substrates by SEM imaging showing many bacteria with destroyed membranes (Figure 13g,i), indicated by the lower viable bacteria counts in the surrounding area, could be attributed to the enhanced surface characteristics of the nanopillar structures. These results are in agreement with previous studies showing exceptional antibacterial properties of nanostructured surfaces fabricated by the HT process [13,14,27]. The observed reduction in bacterial adhesion and biofilm formation on the dual-scale modified titanium surfaces may have clinical implications, particularly in the prevention of peri-implantitis—a multifactorial inflammatory condition primarily driven by biofilm accumulation and a leading cause of late-stage implant failure. Our findings align with previous reports that highlight the significance of surface modifications in controlling peri-implant infections [54,55,56]. These studies have demonstrated that *S. aureus*, due to its strong biofilm-forming ability, plays an important role in the pathogenesis of peri-implantitis [56]. In addition, *E. coli* has been identified in early-stage peri-implant mucositis. Similarly to our study, it was reported that Poly[2-(methacryloyloxy)ethyl choline phosphate]-modified Ti surfaces could disrupt early *E. coli* and *S. aureus* colonization [54].

These nanopillar structures appear to support bacterial killing in adjacent areas, offering a complex but potentially beneficial interaction that could be harnessed in the design of implant surfaces to reduce infection rates and improve implant success in clinical applications. In contrast with the control smooth Ti surfaces, bacteria seem to be able to survive more readily in the surrounding areas, possibly due to a lack of surface modifications that could otherwise disrupt bacterial activity. Overall, these results of the CFU, MTT assays, and SEM imaging indicate that while the 3D-Ti-MS-NS materials with combined micro- and nanostructures support bacterial attachment, they show properties for inducing bacterial killing areas, which are desirable features for implant materials to reduce the risk of infection. Further investigations into the specific mechanisms underlying these effects, such as antimicrobial surface properties or the influence of surface nanostructures on bacterial behavior, are needed to fully understand the interaction between titanium implants and bacterial biofilms. While *S. aureus* and *E. coli* were selected as representative Gram-positive and Gram-negative strains, respectively, we acknowledge that the use of only two bacterial species limits the extent of our antimicrobial evaluation. Peri-implant infections are often polymicrobial in nature, involving complex biofilms that include anaerobes and opportunistic pathogens (e.g., *Candida albicans)*. Future studies could expand the microbial panel and incorporate multispecies biofilm models to provide a more comprehensive understanding of the antimicrobial efficacy under conditions that more closely mimic the in vivo peri-implant environment.

## 5. Conclusions

This study demonstrated that 3D surface modifications on titanium substrates with combined micro- and nanostructures significantly enhance cell viability, osteointegration, and antimicrobial properties compared with smooth Ti surfaces. SEM, EDX, and XRD analyses confirmed the formation of microspheres on 3D-Ti-MS and nanopillars on 3D-Ti-MS-NS surfaces, with the latter exhibiting increased hydrophilicity that is favorable for better cell interactions that are confirmed by the presented results. Dental pulp stem cell viability assays showed that 3D-Ti-MS-NS promoted higher cell growth and membrane integrity, while osteogenic assays confirmed its ability to enhance osteodifferentiation. Additionally, both 3D-Ti-MS and 3D-Ti-MS-NS demonstrated reduced bacterial adhesion and biofilm formation, whereas 3D-Ti-MS-NS showed the ability to mechanically destroy attached bacterial cells, highlighting their potential for preventing implant infections.

In conclusion, 3D-fabricated titanium implants with combined micro- and nanostructures offer significant advantages compared with traditional Ti implants, providing low-cost, fast, and custom-made on-site implants when they are needed. Their additional advancements in terms of biological performance and antimicrobial properties make them promising candidates for the next generation of Ti implants for various applications, specifically for dental implants. Future studies should focus on optimizing surface topography, further enhancing antimicrobial effects through functional coatings, and conducting long-term in vivo studies to assess the clinical efficacy of these materials. Exploring the molecular mechanisms of osteogenesis on 3D-Ti-MS-NS surfaces and validating these findings in clinical trials will be key steps toward ensuring their successful translation to implant applications.

## Data Availability

Data underlying the results presented in this paper may be obtained from the authors upon reasonable request.

## Supplementary Materials

The following supporting information can be downloaded at: https://www.mdpi.com/article/10.3390/jfb16050157/s1, Figure S1: Schematic diagram of wound healing assay; Figure S2. Representative SEM image of Ti (Control). Bar scale is 10 µm, Table S1: The list of specific primers used in the study; Table S2. Growth conditions for activation of bacteria strains used for monomicrobial biofilm formation. Manufacturer of growth medium: *HIMEDIA (India); **ProReady (Serbia). Video S1: Wound scratch healing assay on Ti substrate; Video S2: Wound scratch healing assay on 3D-Ti-MS substrate; Video S3: Wound scratch healing assay on 3D-Ti-MS-NS substrate.
