# Supplementary material for: Combined Effects of Dual-Scale Modified Surface with Micro- and Nanostructures on the Cellular Biocompatibility, Osteoinduction, and Antibacterial Properties of Titanium Implants
Source: J Funct Biomater. 2025 Apr 28;16(5):157. doi: 10.3390/jfb16050157 (PMC12111884; doi:10.3390/jfb16050157)
Supplement: Supplementary file 1 [file jfb-16-00157-s001.zip › jfb-3574839-supplementary data proofs.pdf]

Supporting Information

Combined Effects of Dual-Scale Modified Surface with Micro- and Nanostructures on the Cellular Biocompatibility, Osteoinduction, and Antibacterial Properties of Titanium Implants

Shaheer Maher [1], Nenad L. Ignjatović[2]*, Milos Lazarevic[3], Sanja Petrović[3], Andrijana Žekić[4] and Dušan Losić [5]*

[1] Faculty of Pharmacy, Assiut University, Assiut, 71526, Egypt
[2] Institute of Technical Sciences of the Serbian Academy of Sciences and Arts, Knez Mihailova 35/4, 11000 Belgrade, Serbia
[3] School of Dental Medicine, University of Belgrade, Dr Subotica 8, 11000 Belgrade, Serbia
[4] Faculty of Physics, University of Belgrade, Studentski trg 12, 11000 Belgrade, Serbia
[5] School of Chemical Engineering, The University of Adelaide, Adelaide SA 5005, Australia
* Correspondence: dusan.losic@adelaide.edu.au (D.L.), and nenad.ignjatovic@itn.sanu.ac.rs (N.L.I.)

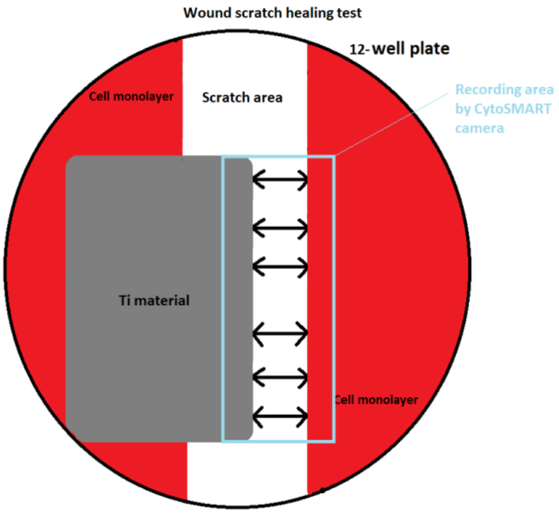

Figure S1. Schematic diagram of wound healing assay.

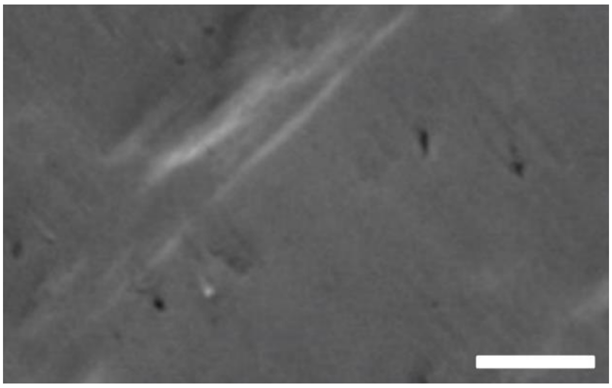

Figure S2. Representative SEM image of Ti (Control). Bar scale is 10µm.

Table S1. The list of specific primers used in the study.

Gene		Primer sequence	Accession No.	Amplicon size (bp)	Annealing temperature (°C)
OCN	Rv	5′CTC ACA CTC CTCGCC CTA TT 3′	NM_199173.6	107	58
	Fw	5′ TTG GAC ACA AAG GCT GCA C 3′			
ALP	Rv	5′ ATG GCA GTG AAG GGC TTC TT 3′	NM_000478.6	99	58
	Fw	5′ CCA CGT CTT CAC ATT TGG TG 3′			
GAPDH	Rv	5′ CCC TGT TGC TGT AGC CAA ATT CGT 3′	NM_002046.7	450	58
	Fw	5′ TCA TGA CCA CAG TCC ATG CCA TCA 3′			

Table S2. Growth conditions for activation of bacteria strains used for monomicrobial biofilm formation. Manufacturer of growth medium: *HIMEDIA (India); **ProReady (Serbia).

**Bacteria strain**	**Solid growth medium**	**Broth (liquid growth medium)**	**Temperature**	**Time**	**Conditions**
Echerichia coli	Endoagar*	Brain Heart infusion broth*	37 °C	24h	Aerobic
Staphylococcus aureus	Columbia agar* with 5% sheep blood**		37 °C	24h	Aerobic

Supplementary video S1: Title: Wound scratch healing assay on Ti substrate.
The video captures a wound healing assay during 48h where DPSCs migrate to the edge of a titanium material. The assay examines the cellular movement and wound closure dynamics in response to the titanium material. Cells actively move towards the scratched area adjacent to the Ti

plate, indicating a healing response. Some cells appear to interact with or adhere near the Ti surface, which could indicate its influence on cellular behavior.

Supplementary video S2: Title: Wound scratch healing assay on 3D-Ti-MS substrate.
The video captures a wound healing assay during 48h where DPSCs migrate to the edge of a 3D-Ti-MS material. The assay examines the cellular movement and wound closure dynamics in response to the titanium material. Cells actively move towards the scratched area adjacent to the 3D-Ti-MS plate, indicating a healing response. Some cells appear to interact with or adhere near the 3D-Ti-MS surface, which could indicate its influence on cellular behavior.

Supplementary video S3: Title Wound scratch healing assay on 3D-Ti-MS-NS substrate.
The video captures a wound healing assay during 48h where DPSCs migrate to the edge of a 3D-Ti-MS-NS material. The assay examines the cellular movement and wound closure dynamics in response to the 3D-Ti-MS-NS material. Cells actively move towards the scratched area adjacent to the 3D-Ti-MS-NS plate, indicating a healing response. Some cells appear to interact with or adhere near the 3D-Ti-MS-NS surface, which could indicate its influence on cellular behavior.
